# *Salmonella enterica* serovar Typhimurium mutants completely lacking the F_0_F_1_ ATPase are novel live attenuated vaccine strains

**DOI:** 10.1016/j.vaccine.2009.10.146

**Published:** 2010-01-22

**Authors:** H. Northen, G.K. Paterson, F. Constantino-Casas, C.E. Bryant, S. Clare, P. Mastroeni, S.E. Peters, D.J. Maskell

**Affiliations:** a Department of Veterinary Medicine, University of Cambridge, Madingley Road, Cambridge CB3 0ES, UK; b Wellcome Trust Sanger Institute, Wellcome Trust Genome Campus, Hinxton, Cambridge CB10 1SA, UK

**Keywords:** *Salmonella* Typhimurium, F_0_F_1_ ATPase, Vaccination

## Abstract

The F_0_F_1_ ATPase plays a central role in both the generation of ATP and the utilisation of ATP for cellular processes such as rotation of bacterial flagella. We have deleted the entire operon encoding the F_0_F_1_ ATPase, as well as genes encoding individual F_0_ or F_1_ subunits, in *Salmonella enteric* serovar Typhimurium. These mutants were attenuated for virulence, as assessed by bacterial counts in the livers and spleens of intravenously infected mice. The attenuated *in vivo* growth of the entire *atp* operon mutant was complemented by the insertion of the *atp* operon into the *malXY* pseudogene region. Following clearance of the attenuated mutants from the organs, mice were protected against challenge with the virulent wild type parent strain. We have shown that the F_0_F_1_ ATPase is important for bacterial growth *in vivo* and that *atp* mutants are effective live attenuated vaccines against *Salmonella* infection.

## 1 Introduction

*Salmonella enterica* is a diverse pathogen classified into >2400 serovars and is the cause of important infections in both humans and livestock. *S. enterica* serovar Typhi (*S*. Typhi) is the causative agent of typhoid fever, a serious systemic disease in humans. It is estimated that there are 22 million cases of typhoid fever annually worldwide, resulting in 200,000 deaths [1,2]. Vaccination against *S*. Typhi is a potentially attractive method of disease control, but current vaccines have significant drawbacks and there is a need for improved versions [3,4]. *S.* *enterica* serovar Typhimurium (*S*. Typhimurium), a common cause of gastroenteritis (salmonellosis) in humans, has added significance because infection of mice with this serovar generates a systemic infection with important similarities to human typhoid fever. This mouse model has been used extensively to study typhoid-like infections [5,6].

The F_0_F_1_ ATPase is a complex of membrane proteins found in eukaryotes and prokaryotes that has been best studied in mitochondria [7,8], chloroplasts [9,10] and *Escherichia coli* [11–13]. It plays a central role in energy transduction, generating ATP from ADP and Pi substrates via oxidative phosphorylation. The synthesis of ATP is driven by the flow of protons into the cell, generating a proton motive force which energises processes such as motility and active transport [14–17]. In *E. coli*, the genes encoding the F_0_F_1_ ATPase are located in a single operon, *atpIBEFHAGDC*, transcribed from a promoter upstream of *atpI* [18–20]. The F_0_ subunit of the ATPase is a hydrophobic membrane-embedded proton channel encoded by genes *atpBEF*. The F_1_ subunit constitutes the catalytic ATPase, encoded by *atpHAGDC* [19,21]. The first gene in the operon, *atpI*, has no defined function and does not appear to form part of the F_0_F_1_ ATPase complex [22]. This genetic organisation is conserved between *E. coli* and *S*. Typhimurium.

A comprehensive identification of genes required for *S*. Typhimurium infection of mice by our laboratory identified mutation of *atpA* as an attenuating lesion [23]. A defined *atpA* deletion mutant was subsequently confirmed to be attenuated for growth *in vivo* and furthermore was found to offer significant protection against subsequent challenge [23]. Here we present a full analysis of the role of the F_0_F_1_ ATPase in *S*. Typhimurium infection and the potential use of mutants in the *atp* operon as live attenuated vaccines.

## 2 Materials and methods

### 2.1 Bacterial strains and growth conditions

The bacterial strains and plasmids used in this study are shown in Table 1. Bacteria were grown at 37 °C in Luria–Bertani (LB) broth or on LB agar. Media were supplemented with antibiotics where stated, at the following concentrations, kanamycin 50 μg/ml, ampicillin 100 μg/ml and chloramphenicol 25 μg/ml. Minimal medium (used to determine carbon source utilisation) consisted of M9 salts (Sigma Dorset UK) supplemented with 0.1 mM CaCl_2_, 1 mM MgSO_4_, 4 μg/ml histidine and the stated carbon source at 0.4% (final w/v).

### 2.2 Construction of mutants and complementation

Oligo-directed mutagenesis (ODM), an adaptation of ET-cloning, was used to replace the target genes on the *Salmonella* chromosome with a kanamycin resistance cassette flanked with FRT regions from pBADkanFRT [24,25]. PCR was used to amplify the kanamycin resistance FRT cassette with 5′ and 3′ 60 bp arms homologous to DNA flanking the target genes (see Table 2 for primer sequences). *S*. Typhimurium LB5010 containing pBADλred was grown in LB broth supplemented with ampicillin to an OD_595_ of 0.25. Arabinose was added to 0.2% (final w/v) to induce *red* gene expression. Cultures were grown to OD_595_ 0.5 and electroporated with the purified ODM PCR product described above. Mutant colonies were selected on LB agar plates supplemented with 50 μg/ml kanamycin. The desired allelic replacement of the target genes was confirmed by PCR (see Table 2 for primer sequences). Mutations in *S*. Typhimurium LB5010 were transduced into SL1344 by bacteriophage P22 as described previously [26] with selection on LB agar plus kanamycin and gene deletions were confirmed to be correct by PCR and sequencing.

The kanamycin resistance FRT cassette was then excised to leave only a 128 bp FRT scar site. Briefly, electrocompetent mutants of SL1344 were transformed with pCP20 [24] grown at 30 °C and then plated onto LB agar containing 100 μg/ml ampicillin. Single colonies were grown in LB at 39 °C (to prevent replication of pCP20) for 6 h then diluted and plated onto LB agar and incubated overnight at 39 °C. Colonies were screened for loss of ampicillin and kanamycin resistance. Excision of the kanamycin resistance FRT cassette was confirmed by PCR and sequencing to be correct. Southern blot using the FRT scar site region as a probe was also used to confirm that the final mutants were as intended. LPS serotype was confirmed by agglutination with anti-04 serotype antiserum using anti-09 antiserum as a negative control (Remel Europe Ltd./Oxoid Ltd., Basingstoke UK).

For complementation of SL1344 *atp*, lacking the entire *atp* operon, PCR was used to amplify the entire *atp* operon from SL1344 fused to a chloramphenicol resistance cassette, from pACYC184. This was inserted into the *malXY* pseudogene region on the *Salmonella* chromosome using ODM with selection on chloramphenicol. Insertion of the *atp* operon into *malXY* was confirmed by PCR and sequencing of the mutated *malXY* junction and by Southern blotting using *atpG* as the probe. In addition to the complemented strain, SL1344 *atp* (*malXY atp* operon^+^), a complementation control strain was also generated, SL1344 *atp* (*malXY* Cm^R^). For this control strain a chloramphenicol resistance cassette was inserted into the *malXY* pseudogene region of SL1344 *atp* to ensure the insertion into the pseudogene had no phenotypic effects.

### 2.3 Growth *in vitro* and succinate utilisation

Cultures in 5 ml of LB broth were incubated overnight with shaking (180 rpm) at 37 °C. Cultures were diluted 1:100,000 into 100 ml of pre-warmed LB broth, and incubated with shaking at 37 °C. Growth was measured by viable count on LB agar plates. Exponential generation times were calculated from growth rates between 4 and 6 h.

To assess the ability to utilise succinate as a sole carbon source wild type and the various *atp* mutants were grown in M9 minimal medium supplemented with 0.4% (w/v) of sodium succinate. Growth was assessed by OD_595_ after 24 and 48 h.

### 2.4 Mouse typhoid model

Inocula were prepared from overnight cultures grown statically in LB broth at 37 °C. Cultures were centrifuged and bacteria were re-suspended in phosphate buffered saline (pH 7.4) to the required concentration. Seven to nine week-old female BALB/c mice (Harlan, Oxon, UK) were inoculated with 200 μl of bacteria suspension via intravenous injection, or they were lightly anaesthetised with halothane and inoculated by oral gavage. Doses of bacteria given were confirmed by viable counts in LB agar. Gene knock-out mice lacking gp91*phox* or IFNγR1 on a C57/BL6j background where originally purchased from Jackson Laboratory (Bar Marbour, ME) and maintained as homozygous matings at the Wellcome Trust Sanger Institute. C57/BL6j age- and sex-matched control mice were purchased from Harlan (Oxon, UK).

At pre-determined time points postinfection animals were killed, spleens and livers removed and homogenised in 5 ml of sterile water in a Stomacher^®^ 80 Lab System (Seward). Bacterial numbers were enumerated via serial dilutions and plating in LB agar. When required, blood was collected via cardiac puncture under terminal anaesthesia.

For vaccination studies, animals were immunised intravenously with 10^5^ CFU or orally with 10^9^ CFU. At these doses, immunising strains did not induce clinical signs, were completely cleared with all mice surviving the infection. At 13 weeks postimmunisation clearance of the bacteria was confirmed by viable counts from spleens and livers. Mice were subsequently re-challenged either intravenously with 10^4^ CFU, or orally with 10^8^ CFU of SL1344. Age-matched unimmunised mice were included for comparison. Viable counts in the target organs were enumerated as detailed above. All work was licensed by the UK Home Office.

### 2.5 Histolopathological analysis

For histopathological analysis, a portion of spleen was fixed in 10% buffered formalin then embedded in paraffin wax. Four 3 μm sections were cut approximately 20–30 μM apart then stained with Haematoxylin and Eosin (H&E). Spleen sections were examined microscopically.

### 2.6 Anti-*Salmonella* antibodies assayed by ELISA

Sonicated SL1344 was used as the ELISA capture antigen to assay anti-*Salmonella* antibodies following vaccination. This was diluted in carbonate coating buffer (1.59 g/l sodium carbonate, 2.93 g/l sodium bicarbonate, pH 8.2) to 1 × 10^6^ bacteria/ml, based on the viable count of the original culture. 100 μl of this antigen solution was used to coat the wells of an ELISA plate (Immunoplates, Nunc, Thermofisher Scientific, Lutterworth, UK) through overnight incubation at 4 °C. Plates were washed with washing buffer (PBS containing 0.05%, w/v, Tween 20) then wells were blocked with 300 μl/well of blocking buffer (PBS containing 1% bovine serum albumin) for 2 h. Serial fivefold dilutions of heat-inactivated mouse serum were prepared in blocking buffer and 100 μl were added to washed plates. Sera from normal mice and known positive sera were included on each plate as negative and positive controls. Plates were incubated for 2 h at room temperature. Total antibody was detected using 100 μl/well of biotinylated goat anti-mouse immunoglobulins (Dako, Ely, UK) diluted 1:1000 in blocking buffer. Subtypes IgG1 and IgG2a were detected using 100 μl/well of biotinylated rat anti-mouse IgG1 or IgG2a antibodies (BD Bioscience, Oxford, UK) diluted 1:500 in blocking buffer. Plates were incubated with secondary antibody for 1 h at room temperature and then washed three times in wash buffer. Then 100 μl/well of streptavidin (BD Bioscience, Oxford, UK), diluted 1:100 in blocking buffer, was added and plates were incubated in the dark for 30 minutes. Plates were then washed and developed with 100 μl TMB substrate solution (BD Bioscience, Oxford, UK) and the reaction stopped with the addition of 50 μl/well of 5N sulphuric acid. Absorbance was read at 450 nm. Data presented are from dilutions of 1:12,500 for total Ig and 1:2500 for Ig subclasses.

### 2.7 Macrophage infection *in vitro*

RAW 264.7 cells were seeded into 96 well plates at a density of 2 × 10^5^ cells/well in RPMI medium (Sigma Dorset, UK) supplemented with 10% FCS and 2 mM l-glutamate. Plates were seeded the evening before infection and incubated throughout at 37 °C with 5% CO_2_. For the bacterial inoculum, overnight cultures were diluted 1:10 into fresh LB broth and incubated for 2 hr at 37 °C with shaking. Bacteria were collected by centrifugation, re-suspended in PBS and diluted in tissue-culture medium to the required concentration. Bacteria were added to host cells and incubated at 37 °C 5% CO_2_ for 2 h. The monolayer was washed twice in pre-warmed PBS and medium containing 50 μg/ml gentamicin was added to kill extracellular bacteria. Following incubation for 1 h host cells were washed twice with PBS and medium containing 10 μg/ml gentamicin was added for the remainder of the experiment. Intracellular bacteria were enumerated by serial dilution and plating on LB agar following lysis of host cells with 0.5% Triton 100×. Following the manufacturer's instructions, the Cytotox96 assay kit (Promega, Southampton, UK) was used to determine the relative viability of host cells after infection.

### 2.8 Statistical analysis

Statistical analysis was performed using Student's *t*-test or one-way ANOVA with Bonferroni correction. *P* ≤ 0.05 was considered significant.

## 3 Results

### 3.1 Growth of *S*. Typhimurium *atp* mutants *in vitro*

Deletion mutants were generated in SL1344 that lacked the entire *atp* operon or the F_0_ or F_1_ components only. When grown in LB broth the various *atp* mutants all had similar generation times in comparison with SL1344. These were 29.72 (±0.78) min for SL1344, 32.22 (±1.90) min for SL1344 F_0_, 33.12 (±1.06) min for SL1344 F_1_ and 29.24 (±0.85) min for SL1344 *atp* (all mean ± SEM from 3 replicates). However, final viable bacterial counts of overnight cultures were consistently lower in the various *atp* mutants compared to SL1344. The viable counts in 24 hr cultures were log_10_ 9.69 CFU (±0.08) for SL1344, log_10_ 9.19 CFU (±0.04) for SL1344 F_0_, log_10_ 9.21 CFU (±0.16) for SL1344 F_1_ and log_10_ 9.29 CFU (±0.09) for SL1344 *atp* (all mean ± SEM from 3 replicates), although these differences were only statistically significant between SL1344 and SL1344 F_0_.

As seen with mutations in the *atp* operon in *E. coli* [27], *Bacillus subtilis* [28] and *S*. Typhimurium [29] all our *atp* mutants were unable to utilise succinate as a sole carbon or energy source. The three *atp* mutants showed no growth after 24 or 48 h, as measured by OD_595_. The *atp* mutants had OD_595_ readings of 0.001 (±0.001) for SL1344 *atp*, 0.0015 (±0.0005) for SL1344 F_0_ and 0.0015 (±0.0005) for SL1344 F_1_ at 48hrs, whereas SL1344 showed visible growth at both 24 and 48 h, with OD_595_ readings of 0.0335 (±0.01) and 0.374 (±0.07) respectively (all mean ± SEM from 3 replicates).

### 3.2 SL1344 *atp* mutants are attenuated in a mouse model of typhoid fever

Previous studies have shown that individual gene deletions or transposon insertions in the *atp* operon attenuate *S*. Typhimurium in both mice and chickens [23,29,30] but attenuation following deletion of the whole operon or individual subunits has not been tested.

**Fig. 1:**
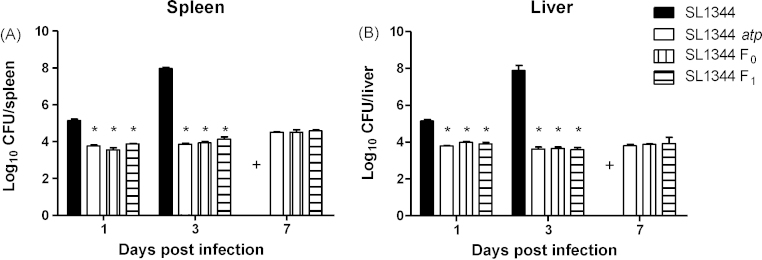
Bacterial counts in spleens and livers following intravenous infection with various *atp* mutants or SL1344. Female BALB/c mice were infected intravenously with 10^5^ CFU of *S*. Typhimurium SL1344, SL1344 *atp*, SL1344 F_0_ or SL1344 F_1_. Bacterial numbers in the spleens (A) and livers (B) were enumerated. Data are presented as mean log_10_ CFU ± SEM (*n* = 4), representative of two experiments giving similar results. (+) SL1344 infected mice were not included on day 7 as mice would not survive to this time point. *P ≤ 0.05 compared to SL1344.

To assess the level of attenuation caused by the deletion of the F_0_ or F_1_ subunits, or the entire *atp* operon, BALB/c mice were infected intravenously with 10^5^ CFU of SL1344, SL1344 F_0_, SL1344 F_1_ or SL1344 *atp*. Bacterial loads in the spleens and livers were enumerated at the time points shown (Fig. 1). In both spleens and livers, bacterial counts were significantly lower in mice infected with the various *atp* mutants in comparison with those infected with SL1344. The three *atp* mutants showed little net bacterial growth between days 1 and 3 postinfection whereas bacterial loads in mice infected with SL1344 increased by nearly 3 logs over the same period. By day 7 the various *atp* mutants showed no significant bacterial growth, with counts similar to those at day 3, whereas mice infected with SL1344 would have been dead by this time point.

### 3.3 Intravenous immunisation with *Salmonellaatp* mutants confers protection against subsequent re-challenge

**Fig. 2:**
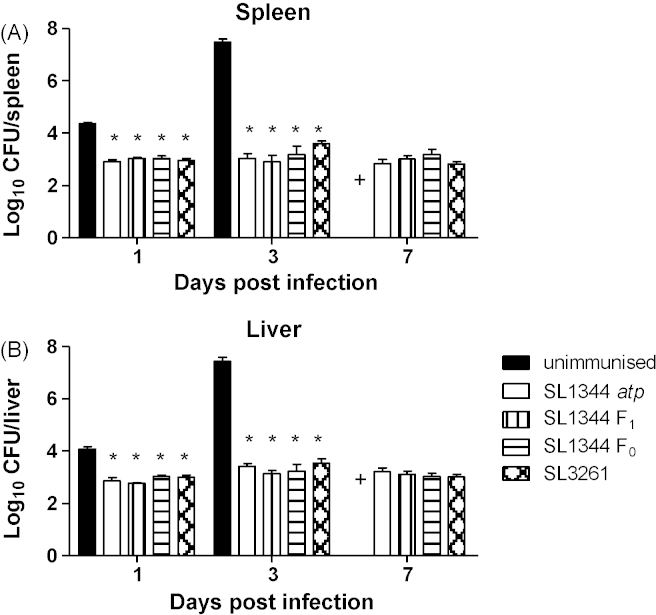
Protection following intravenous vaccination with various *atp* mutants or SL3261. Female BALB/c mice were immunised intravenously with 10^5^ CFU of SL1344 *atp*, SL1344 F_0_, SL1344 F_1_ or SL3261. After 13 weeks, following clearance of the immunising strains, immunised and age-matched unimmunised mice were challenged intravenously with 10^4^ CFU of SL1344. Bacterial numbers in the spleens (A) and livers (B) were enumerated. Data are presented as mean log_10_ CFU ± SEM (*n* = 4), representative of two experiments giving similar results. (+) Unimmunised mice were not included on day 7 as mice would not survive to this time point in contrast to immunised mice which all did survive until then. *P < 0.05 compared to unimmunised mice.

Following immunisation with the three *atp* mutants, mice were re-challenged intravenously with SL1344 (Fig. 2). The wild type infection grew rapidly as expected in unimmunised control mice whereas mice immunised with the *atp* mutants had significantly lower bacterial counts in spleens and livers at days 1 and 4 postinfection. Bacterial counts were comparable between the animals immunised with the different *atp* mutants and with mice immunised with the well-characterised *aroA* mutant vaccine strain, SL3261. Therefore SL1344 F_0_, SL1344 F_1_ and SL1344 *atp* were all protective against subsequent challenge. Since all three *atp* mutants behaved the same in terms of attenuated growth *in vivo* and protection against subsequent infection, SL1344 *atp* was selected for further characterisation.

### 3.4 Complementation of SL1344 *atp* with the *atp* operon restores virulence

**Fig. 3:**
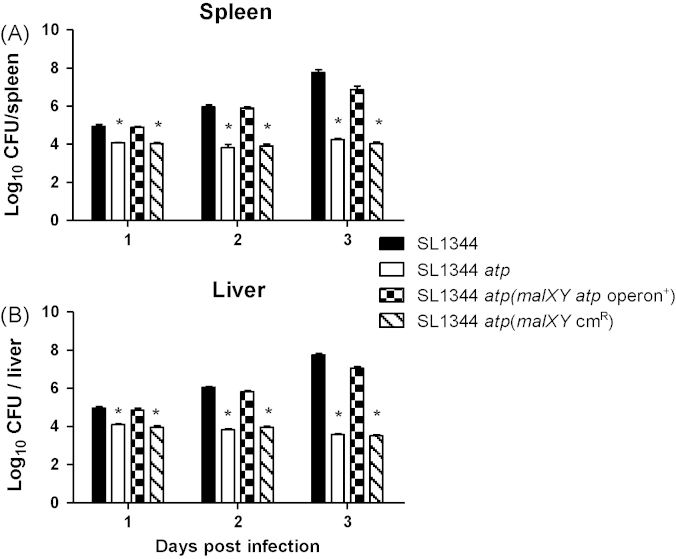
Bacterial counts following intravenous infection with SL1344, SL1344 *atp*, SL1344 *atp* (*malXY atp* operon^+^) or SL1344 *atp* operon (*malXY* Cm^R^). Female BALB/c mice were infected intravenously with 10^5^ CFU of SL1344, SL1344 *atp*, SL1344 *atp* (*malXY atp* operon ^+^) or SL1344 *atp* (*malXY* Cm^R^). Bacterial numbers in the spleens (A) and livers (B) were enumerated. Data are represented as mean log_10_ CFU ± SEM (*n* = 4), representative of two experiments giving similar results. *P ≤ 0.05 significant compared to SL1344.

To confirm that the attenuation of SL1344 *atp* was specifically due to the deletion of the *atp* operon, SL1344 *atp* was complemented by insertion of the whole *atp* operon fused to a chloramphenicol resistance cassette into the *malXY* pseudogene region to generate strain SL1344 *atp* (*malXY* *atp* operon^+^). BALB/c mice were infected intravenously with 10^5^ CFU of SL1344, SL1344 *atp*, SL1344 *atp* (*malXY* *atp* operon^+^) and SL1344 *atp* (*malXY* Cm^R^). The complemented strain, SL1344 *atp* (*malXY atp* operon^+^) displayed a wild type-like phenotype with increased bacterial loads in livers and spleens relative to SL1344 *atp* at days 1, 2 and 3 postinfection (Fig. 3). Insertion of the chloramphenicol resistance cassette into the *malXY* region in strain SL1344 *atp* (*malXY* Cm^R^) had no effect on bacterial counts compared to SL1344 *atp* (Fig. 3).

### 3.5 SL1344 *atp* is not impaired for infection of macrophages *in vitro*

**Fig. 4:**
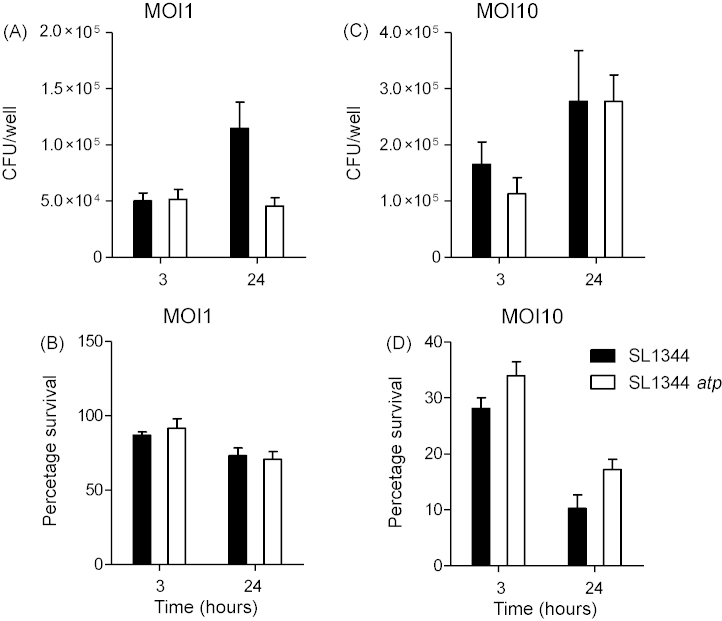
Intracellular survival of SL1344 or SL1344 *atp* in RAW264.7 cells. Intracellular bacteria (A and C) and macrophage survival (B and D) were determined at 3 and 24 h postinfection, with MOIs of I (A and B) and 10 (C and D). Macrophage survival expressed as percentage of survival compared to uninfected cells. Data are represented as mean ± SEM from three experiments each performed in triplicate and giving similar results.

Survival and replication of SL1344 and SL1344 *atp* were assessed in the RAW 264.7 murine macrophage-like cell line. Host cells were infected at MOIs of 1 and 10 and intracellular bacterial counts and macrophage survival were determined at 3 and 24 h postinfection. At both MOIs and at both time points intracellular bacterial viable counts and macrophage survival were similar after infection with SL1344 or SL1344 *atp* with no statistically significant difference between the two strains (Fig. 4).

### 3.6 NADPH oxidase (phox) and IFNγ contribute to control of SL1344 *atp* infection

**Fig. 5:**
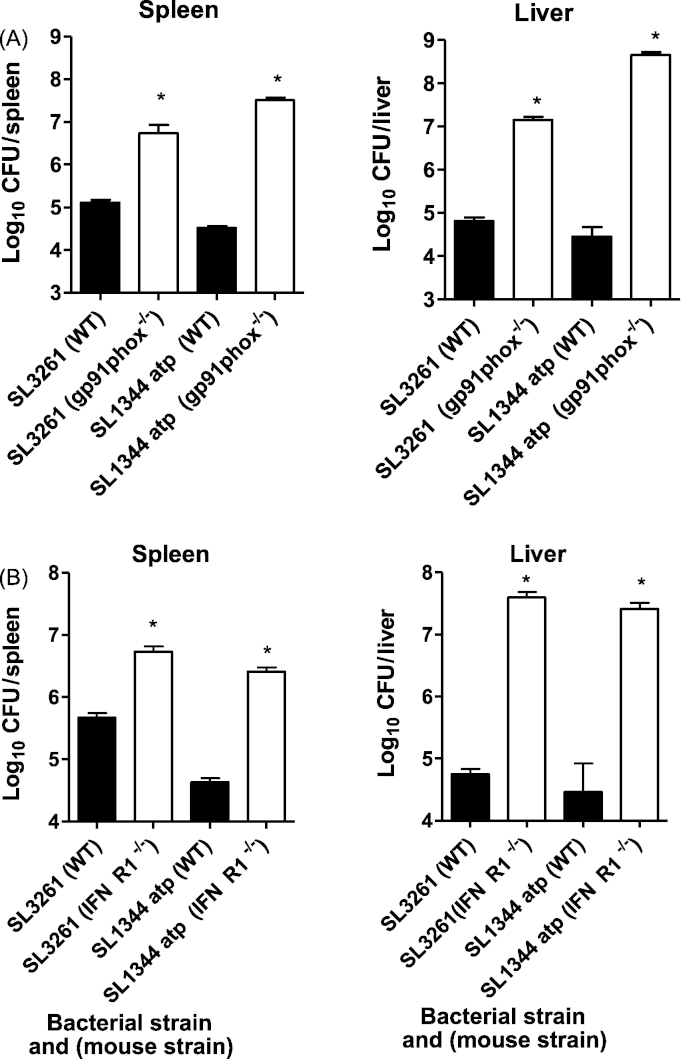
Bacterial counts in gp91*phox* knock-out, IFNγR1 knock-out or wild type C57/BL6 mice. Mice were infected intravenously with 10^5^ CFU of SL1344 *atp* or SL3261. Bacterial numbers in the spleens and livers of gp91*phox* knock-out or wild type C57/BL6 mice were enumerated on day 4 postinfection (3–5 mice per point). Bacterial counts in the spleens and livers of IFNγR1 knock-out or wild type C57/BL6 mice were enumerated on day 9 postinfection (4–5 mice per point). Data are presented as mean log_10_ CFU ± SEM. *P < 0.05 knockout mice compared to wild type mice for each bacterial strain.

To begin to define the immunological components required to control infection with SL1344 *atp* and to assess the potential use of SL1344 *atp* immunisation in immunocompromised individuals, two gene knock-out mouse strains and their respective wild types were infected with SL1344 *atp*. Following infection with SL1344 *atp*, gp91*phox*^−/−^ mice had significantly increased bacterial loads in spleens and livers relative to wild type mice (Fig. 5A) as did mice lacking IFNγR1 (Fig. 5B). These data indicate a significant role for NADPH oxidase and IFN*γ* in controlling bacterial proliferation following infection with SL1344 *atp*. Similarly, both immune components were needed for control of SL3261 replication (Fig. 5).

### 3.7 Intravenous immunisation with SL1344 *atp* confers protection against subsequent oral re-challenge

**Fig. 6:**
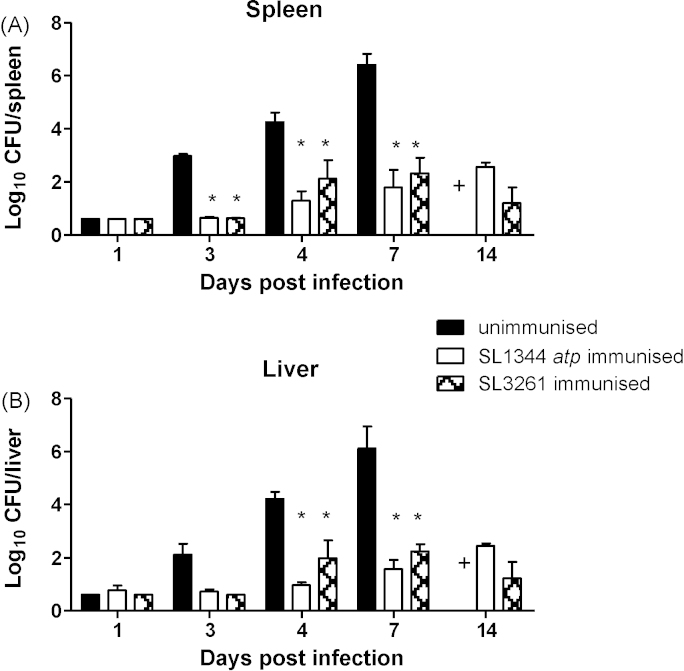
Protection following intravenous vaccination with SL1344 *atp* or SL3261. Female BALB/c mice were immunised intravenously with 10^5^ CFU of SL1344 *atp* or SL3261. After 13 weeks, following clearance of the immunising strains, immunised and age-matched unimmunised mice were challenged orally with 10^9^ CFU SL1344. Bacterial counts in the spleens (A) and livers (B) were enumerated. Data are presented as mean log_10_ CFU ± SEM (*n* = 4), representative of two experiments giving similar results. (+) Unimmunised mice were not included on day 14 as mice would not survive to this time point in contrast to immunised mice which all did survive until then. *P < 0.05 immunised compared to unimmunised mice.

SL1344 *atp* was assessed for its ability to protect against subsequent oral re-challenge (Fig. 6). Again, the wild type challenge grew rapidly, as expected, in unimmunised mice whereas mice immunised with SL1344 *atp* had significantly reduced bacterial counts in spleens on days 3, 4 and 7 and in livers on days 4 and 7 postinfection (Fig. 6). Similar levels of protection were observed between SL1344 *atp* and SL3261-immunised mice (Fig. 6). Therefore, SL1344 *atp* is protective against subsequent oral challenge and this protection is as effective as immunisation with SL3261.

### 3.8 SL1344 *atp* is protective following oral immunisation

**Fig. 7:**
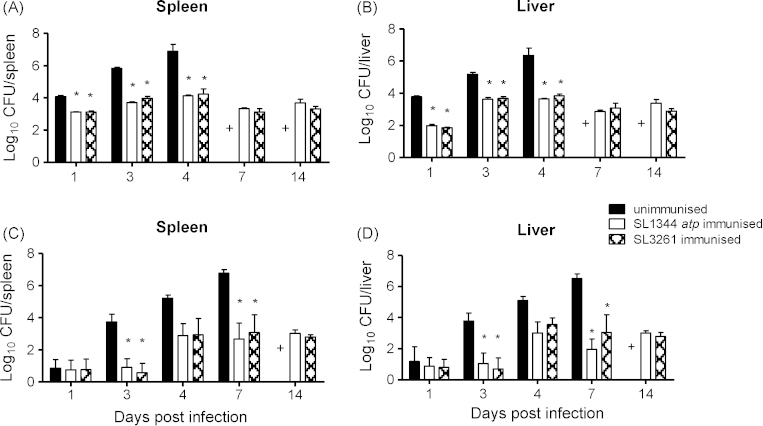
Protection following oral vaccination with SL1344 *atp* or SL3261. Female BALB/c mice were immunised orally with 10^9^ CFU of SL1344 *atp* or SL3261. After 13 weeks, following clearance of the immunising strains, immunised and age-matched unimmunised mice were challenged either intravenously (A and B) with 10^4^ of CFU SL1344 or orally (C and D) with 10^9^ CFU of SL1344. Bacterial counts in spleens and livers were enumerated. Data are presented as mean log_10_ CFU ± SEM (*n* = 4), representative of two experiments giving similar results. (+) Unimmunised mice were not included on days 7 and 14 during intravenous challenge and day 14 during oral challenge as mice would not survive to this time point in contrast to immunised mice which all did survive until these times. *P < 0.05 immunised compared to unimmunised mice.

SL1344 *atp* was further assessed for protection following oral immunisation, given that this would be the preferred route of immunisation with a live attenuated vaccine. The wild type infection grew as expected in unimmunised mice whereas those immunised with SL1344 *atp* had significantly lower bacterial counts in spleens and livers after being re-challenged intravenously (Fig. 7A and B). Little net bacterial growth was observed in challenged SL1344 *atp* immunised mice, with similar levels of bacteria seen over 14 days. Following oral re-challenge, SL1344 *atp* immunised mice showed reduced bacterial counts on days 3 and 7 postinfection relative to unimmunised mice (Fig. 7C and D). Furthermore, bacterial numbers following SL1344 *atp* oral immunisation were comparable to those seen in SL3261-immunised mice regardless of the re-challenge route. The SL1344 *atp* mutant is therefore protective following oral administration and is as effective as SL3261 as a vaccine.

### 3.9 Antibody responses following SL1344 *atp* immunisation

**Fig. 8:**
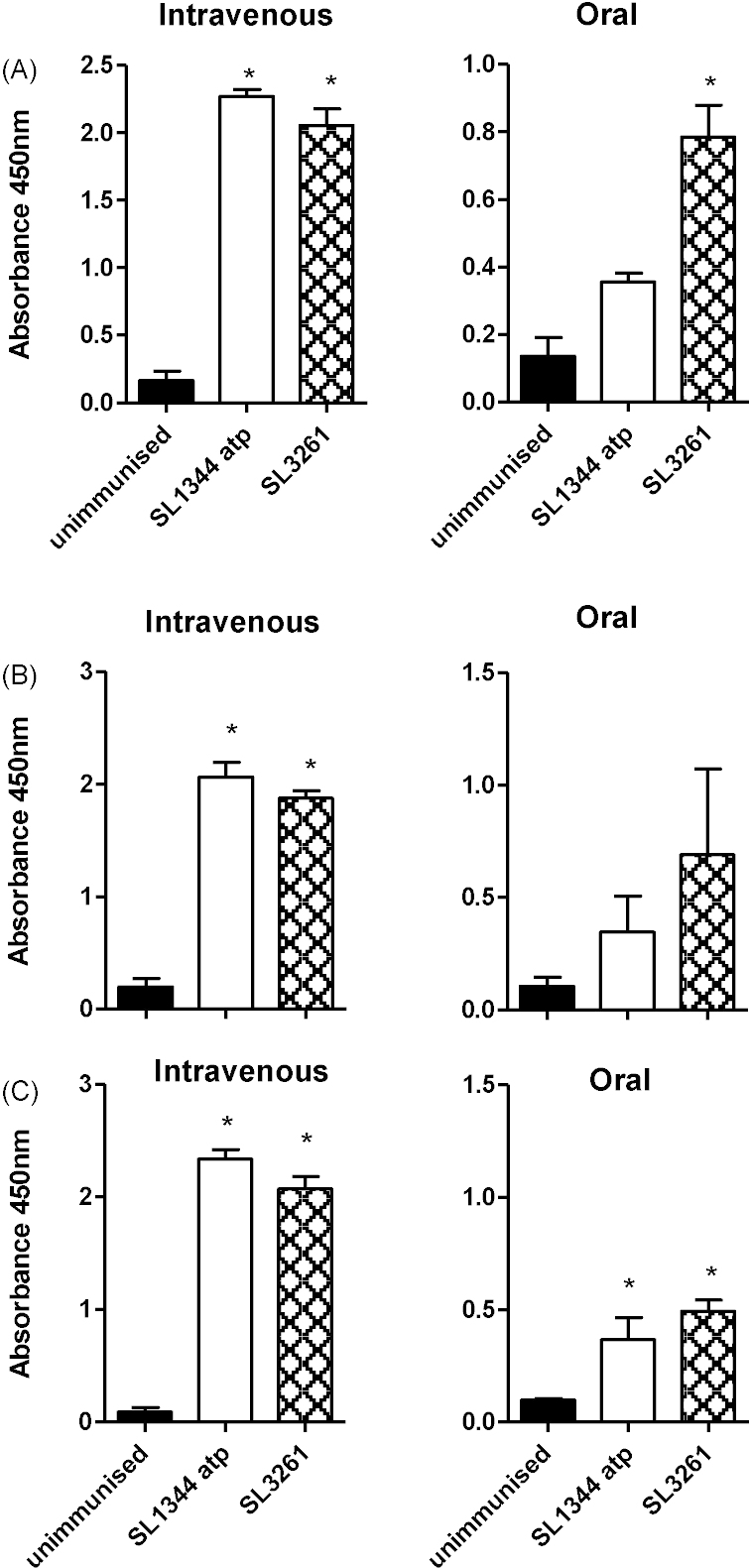
Anti-*Salmonella* antibody levels following vaccination with SL1344 *atp* or SL3261. Pooled sera from 3 to 4 mice taken 11 weeks after intravenous immunisation with 10^5^ CFU or oral immunisation with 10^9^ CFU of SL1344 *atp* or SL3261. Total Ig levels (A) following intravenous or oral immunisation. IgG subclasses IgG1 (B) and IgG2a (C) assayed following intravenous or oral immunisation. *P ≤ 0.05 comparing SL1344 *atp* and SL3261 with unimmunised controls.

Pooled sera from mice immunised intravenously and orally were assayed for antibodies specific for *S*. Typhimurium. Mice intravenously immunised with SL1344 *atp* had significantly higher levels of total antibody against *S.* Typhimurium than unimmunised mice (Fig. 8A). Levels of total antibody in mice intravenously immunised with SL1344 *atp* were comparable to those elicited in SL3261-immunised mice. Total antibody levels following oral immunisation were lower than those seen in intravenously immunised animals, however SL1344 *atp* immunised mice showed higher levels of total antibody compared to unimmunised mice although this did reach statistical significance. Compared with SL3261-immunised mice the antibody levels were lower in SL1344 *atp* immunised mice although this was not statistically significant.

The humoral immune response was further characterised with the determination of IgG subclass levels elicited following immunisation with SL1344 *atp* (Fig. 8B and C). In comparison with unimmunised mice, levels of 1gG21 and IgG2a were higher in SL1344 *atp* immunised mice regardless of immunisation route and SL1344 *atp* immunised mice elicited similar levels of both IgG subclasses in comparison to SL3261.

### 3.10 Reduced splenomegaly following intravenous immunisation with SL1344 *atp* compared to SL3261

**Fig. 9:**
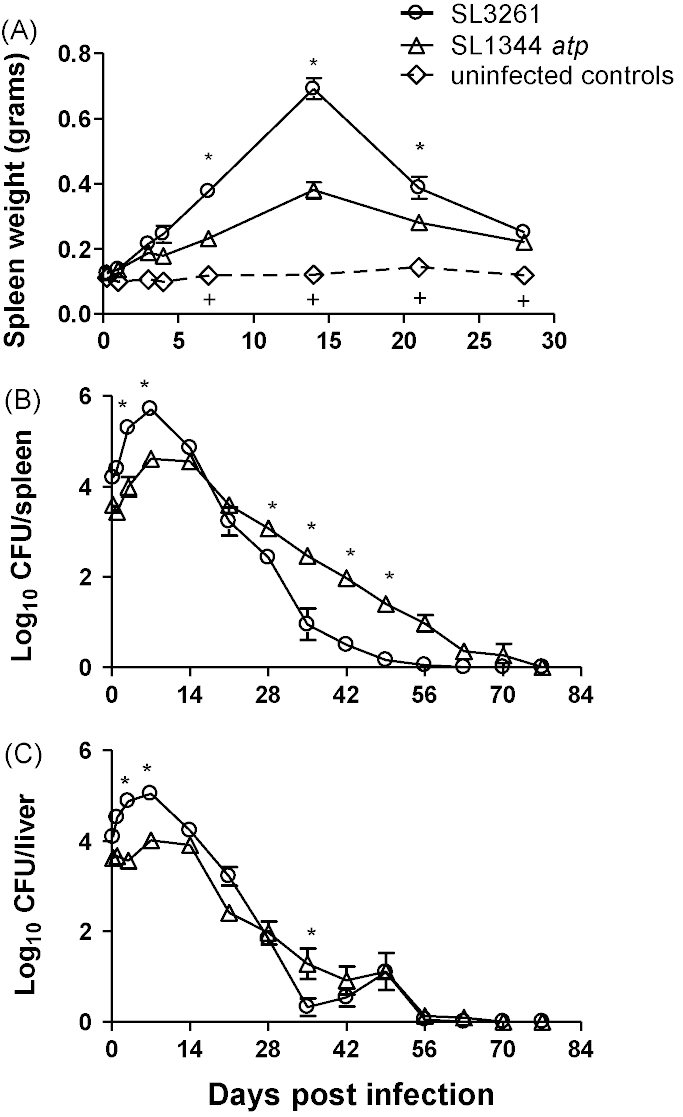
Spleen weight and bacterial counts following vaccination with SL1344 *atp* or SL3261. Female BALB/c mice were immunised intravenously with 10^5^ CFU of SL1344 *atp* or SL3261 and age-matched unimmunised controls were included for spleen weight comparisons. Spleens were weighted (A) and bacterial counts in spleens (B) and livers (C) were enumerated. Data are presented as mean grams ± SEM for spleen weight or mean log_10_ CFU ± SEM for bacterial count (*n* = 4–8). Data are pooled from two experiments giving similar results. ^+^*P* ≤ 0.05 uninfected compared with both infected groups, *P ≤ 0.05 SL1344 *atp* infected mice compared to SL3261 infected mice.

To assess the level of splenomegaly induced following intravenous immunisation with SL1344 *atp* and SL3261, mice were intravenously immunised with 10^5^ CFU and spleen weights were measured along with bacterial viable counts (Fig. 9). In comparison with uninfected age-matched mice, a significant increase in spleen weight was observed in mice immunised with both SL1344 *atp* and SL3261 on days 7, 14, 21 and 28 postinfection (Fig. 9A). In addition, SL3261-immunised mice also showed a significant increase in spleen weight relative to uninfected age-matched mice on days 3 and 4 postinfection. Spleen weights of mice immunised with SL3261 were significantly increased relative to those immunised with SL1344 *atp* on days 7, 14 and 21 postinfection (Fig. 9A). The reduced splenomegaly following immunisation with SL1344 *atp* compared to SL3261, corresponded with lower splenic bacterial counts of SL1344 *atp* which may contribute to the reduced pathology (Fig. 9A and B). Although spleen weights were similar from day 28 onwards in all immunised mice, bacterial counts in the spleens were significantly greater in mice immunised with SL1344 *atp* relative to those immunised with SL3261, from days 28 to 56 postinfection. At 63 days postinfection spleen weights of both immunised groups decreased to a similar level as uninfected controls (data not shown). However SL1344 *atp* immunised mice did not clear bacteria from the spleen until day 77 postinfection, whereas SL3261-immunised animals cleared bacteria at day 63.

In contrast, both SL3261 and SL1344 *atp* immunised mice showed no significant change in liver weight compared with unimmunised controls (data not shown). SL3261 and SL1344 *atp* were both cleared from the livers of immunised mice by day 56 (Fig. 9C).

Histopathological analysis of H&E-stained sections from the spleens of SL3261-immunised mice showed the presence of granulomatous inflammation and areas of pyogranulomatous inflammation with necrosis on day 7 postinfection. In addition SL3261-immunised mice displayed large amounts of lymphoid hyperplasia in conjunction and lymphoid coalescence, resulting in the inability to distinguish red and white pulp areas. These effects were still evident on day 14 postinfection, albeit reduced compared to day 7. At both time points, but especially at day 7, SL1344 *atp* immunised mice displayed much reduced histopathological effects relative to those immunised with SL3261 (data not shown).

## 4 Discussion

We have examined the role of the F_0_F_1_ ATPase in *S*. Typhimurium infection and shown that mutants in this protein complex have potential as live attenuated vaccine strains. The *atpA* gene has previously been identified by our laboratory as part of a screen of transposon mutants, as being required by *S*. Typhimurium for infection of mice [23]. A defined *atpA* mutant was used subsequently to confirm the role of the F_0_F_1_ ATPase in infection, and was also able to confer protection against subsequent wild type challenge. This agrees with previous data showing a role for the F_0_F_1_ ATPase in *Salmonella* infections of mice and chickens [29,30]. We have further characterised the role of the F_0_F_1_ ATPase by comparison of defined non-polar mutants lacking the entire *atp* operon or the F_0_ or F_1_ subunits in SL1344. This is a significant advance on previous work which used undefined or potentially polar mutations. Likewise, the use of *atp* mutants as vaccine strains has not been examined in detail. Our mutants were characterised with respect to their growth *in vitro* and in the mouse model of typhoid fever. All mutants grew as well as SL1344 in LB broth although they reached a slightly lower bacterial cell density at stationary phase. Unlike SL1344, the various *atp* mutants were unable to utilise succinate when it was supplied as the sole carbon source. This inability to use succinate for growth has been shown before for *atp* mutants in *E. coli*, *S.* Typhimurium and *B. subtilis* [27–29].

In the mouse typhoid model, all three *atp* mutants were significantly attenuated for growth with bacterial counts in the spleens and livers of infected mice much lower than those in the organs of mice infected with SL1344. The three *atp* mutants had similar bacterial counts *in vivo* indicating that they were all attenuated to a similar degree and that the two components, F_0_ and F_1_, are equally important for growth *in vivo* with neither subunit contributing to infection independently of the other. This work is the first direct comparison of the relative roles in infection of the two subunits. Our previous demonstration that immunisation with SL1344 *atpA* conferred protection against subsequent SL1344 challenge [23], prompted comparison of the protective efficacy of the *atp* mutant strains generated in this study. All three *atp* mutants protected against SL1344 challenge and did so to a similar degree as the prototype live attenuated vaccine strain SL3261. Given that the three *atp* mutants behaved similarly in terms of attenuation and protection, SL1344 *atp*, lacking the genes encoding the entire *atp* operon was selected for further characterisation. This mutant has the potential advantage of not displaying artefact phenotypes caused by the presence of non-functional F_0_F_1_ ATPase components. Importantly, complementation of SL1344 *atp* with the *atp* operon restored bacterial growth *in vivo* to wild type levels confirming the phenotype was due to the specific deletion of the *atp* operon and not due to secondary mutations. SL1344 *atp* elicited significant protection against virulent challenge when delivered orally, which is likely to be the preferred route of vaccine administration. In addition it was protective against oral challenge, which is the natural route of infection. Furthermore, mice immunised with SL1344 *atp* generated an anti-*Salmonella* antibody response with antibody levels similar to those of SL3261-immunised animals. Of particular note is the production of IgG2a antibodies which are known to play an important role in the rapid clearance of *Salmonellae* through complement activation and the promotion of phagocytosis by macrophages [31–33].

Immunisation with both SL3261 and SL1344 *atp* caused splenomegaly as evidenced by increased spleen weights compared to unimmunised controls. However, the increase in spleen weight was significantly reduced in mice immunised with SL1344 *atp* versus SL3261. This was further examined via histopathological analysis of H&E-stained spleen sections. Consistent with the differences in spleen weights following immunisation, SL1344 *atp* immunised mice showed reduced inflammation and reactogenicity compared to mice immunised with SL3261. This reduction in splenomegaly following SL1344 *atp* immunisation may be a potential benefit of immunisation with SL1344 *atp*.

The ability to infect host macrophages and survive within them is a key process in *Salmonella* infection and mutants impaired in this property are typically attenuated in the mouse model [34]. The ability of SL1344 *atp* to infect and grow within RAW cells was not impaired compared to SL1344. The attenuated growth *in vivo* of SL1344 *atp* is therefore not due to an inherent defect in the infection of and growth within host macrophages. This agrees with previous data showing various *Salmonella* *atp* mutants had no significant deficiency in intracellular survival [29,30]. However, this finding does not exclude the possibility of a defect in this property being manifested specifically *in vivo* where conditions are likely to be very different from those *in vitro*.

Understanding the components of the immune system required to control infection and generate protection following immunisation with live attenuated vaccine strains is of interest as it may offer the potential to enhance immunogenicity and reduce reactogenicity. It also has significance for the use of these strains in immunocompromised hosts. Therefore, IFNγR1^−/−^ and *gp91* phox ^−/−^ counterparts along with their wild type C57BL/6 mice were infected with SL1344 *atp*. These gene knock-out mice are of particular interest as they represent immune defects found in humans. Genetic deficiencies in the NADPH oxidase system (phox) manifest as chronic granulamatous disease [35], while deficiencies in IFNγ activity lead to increased susceptibility to bacterial and fungal infections, particularly with mycobacteria [36,37]. Both NADPH oxidase and IFNγ were required to control SL1344 *atp* infection with bacterial counts in livers and spleens significantly higher in the absence of these host defence mechanisms. A similar effect was seen in mice infected with SL3261. These data are perhaps not surprising given the central role of both NADPH oxidase and IFNγ in the control of *S.* Typhimurium infection in mice [38–40]. However, it is still valuable to compare different vaccine strains in different gene knock-out mice since the mechanisms controlling infection are known to differ between vaccine strains [41,42]. For instance, while IFNγ is required to control infection with SL3261 as shown here and by Vancott et al. [41] it is dispensable for control of infection with a *phoP* mutant.

In summary, we have investigated the role of the F_0_F_1_ ATPase in *S*. Typhimurium infection and shown that this protein complex makes a significant contribution to bacterial growth *in vivo*. Furthermore, mutants lacking the *atp* operon have potential utility as novel live attenuated vaccine strains against *Salmonella* infection.

## Figures and Tables

**Table 1:** *Salmonella* strains and plasmids used in this study.

Bacterial strain or plasmid	Description	References
SL1344	Wild type parent for this work, mouse-virulent strain of *S.* Typhimurium	[43]
LB5010	*S.* Typhimurium. LT2 *galE* mutant, r^−^ m^+^	[44]
SL3261	*aroA* mutant of SL1344. Attenuated in mice. Well-characterised vaccine strain	[43,45]
SL1344 *atp*	*atpIBEFHAGDC* deletion mutant in SL1344	This study
SL1344 F_0_	*atpBEF* deletion mutant in SL1344	This study
SL1344 F_1_	*atpHAGDC* deletion mutant in SL1344	This study
SL1344 *atp* (*malXYatp* operon^+^)	SL1344 *atp* complemented by insertion of *atp* operon into the *malXY* pseudogene region	This study
SL1344 *atp* (*malXY* Cm^R^)	SL1344 *atp* with chloramphenicol resistance cassette inserted into the *malXY* pseudogene region	This study

pBADλred	Plasmid expressing *exo bet gam* genes from bacteriophage lambda	[46]
	*Exo bet gam* genes as *Nco*I-*Hind*III fragment in pBAD/HisA (invitrogen), Amp^R^	
pCP20	Plasmid expressing Flpase genes from *Saccharomyces cerevisiae*. *ClaI-XbaI* fragment of pMMC6 in pHSG415, Amp^R^, Cm^R^	[24]
	Replicates poorly above 37 °C, stably inherited at 30 °C	
pBADkanFRT	pBADTOPO (Invitrogen, Paisley, UK) containing kanamycin cassette flanked by FRT sites. Kan^R^, Amp^R^	[46]

**Table 2:** Primers used in this study.

Name	Sequence 5′–3′	Use
Op ODM For	ttgagaggataaaaaaaaaccagtccgcaatcagactggttttatgctttcaagccggtgtagggataggcttaccttcaagctc	Generation of construct to delete *atp* operon*.* Kanamycin FRT cassette sequence underlined
Op ODM Rev	cttaaagaacgttttatacgacacgcggcatacctcgaagtgagcaggagtaaaaacgtgatgacgatgacaagctccccctttcg	Generation of construct to delete *atp* operon. Kanamycin FRT cassette sequence underlined
F_0_ ODM For	aagctgctttggcgtaggggcgagctaccgtaacaaattcagacatcagcccctccctcctagggataggcttaccttcaagctc	Generation of construct to delete F_0_ region, *atpBEF*. Kanamycin FRT cassette sequence underlined
F_0_ ODM Rev	ggtgctggtggttcagatactggcgccggctgtaattaacaacaaagggtaaaaggcatcatgacgatgacaagctccccctttcg	Generation of construct to delete F_0_region, *atpBEF*. Kanamycin FRT cassette sequence underlined
F_1_ ODM For	ttgagaggataaaaaaaaaccagtccgcaatcagactggttttatgctttcaagccggtgtagggataggcttaccttcaagctc	Generation of construct to delete F_1_region, *atpHAGDC*. Kanamycin FRT cassette sequence underlined
F_1_ ODM Rev	agctgctaacagcgacatcgtggataaacttgtcgctgaactgtaaggagggaggggctgatgacgatgacaagctccccctttcg	Generation of construct to delete F_1_region, *atpHAGDC*. Kanamycin FRT cassette sequence underlined
Op test For	cacaatgtgcagatgccaatgacag	Confirmation of *atp* operon deletion
Op test Rev	atgtttaatgtgtgatctggtgcac	Confirmation of *atp* operon deletion.
F_0_ test For	ggttctgaccgttttcgtctaactg	Confirmation of F_0_ region, *atpBEF* deletion
F_0_ test Rev	atgatatttgcctggcgtcacc	Confirmation of F_0_ region, *atpBEF* deletion
F_1_ test For	aatgacatagtaataatccctcat	Confirmation of F_1_ region, *atpHAGDC* deletion.
F_1_ test Rev	cgcgctcagatcctggacgaagcca	Confirmation of F_1_ region, *atpHAGDC* deletion
F_0_F_1_comp For	ccgcaggttcagtcggtaaaagatgaaatggttggcctgatgaataccgttcaggcataacgacgcggcttgtgttaaaaatcgac	Generation of construct to insert the *atp* operon into the *malXY* pseudogene region. Homologous *malX* sequence underlined
F_0_F_1_comp Rev	ctacgtacaccatgtcccgcgtcggtcaacttcctgtgaaaaatcgaacatatcccttccgcttattatcacttattcaggcg	Generation of construct to insert the *atp* operon into the *malXY* pseudogene region. Homologous *malY* sequence underlined
F_0_F_1_ test For	catcgtgagtctggacaactgcat	Confirmation of *atp* operon insertion into *malXY* pseudogene region
F_0_F_1_ test Rev	ataatcccactacgtacaccatgtc	Confirmation of *atp* operon insertion into *malXY* pseudogene
